# Childhood fish oil supplementation modifies associations between traffic related air pollution and allergic sensitisation

**DOI:** 10.1186/s12940-018-0370-5

**Published:** 2018-03-27

**Authors:** Anna L. Hansell, Ioannis Bakolis, Christine T. Cowie, Elena G. Belousova, Kitty Ng, Christina Weber-Chrysochoou, Warwick J. Britton, Stephen R. Leeder, Euan R. Tovey, Karen L. Webb, Brett G. Toelle, Guy B. Marks

**Affiliations:** 1 MRC-PHE Centre for Environment and Health, Department of Epidemiology and Biostatistics, School of Public Health, Imperial College London, St Mary’s Campus, Norfolk Place, London, W2 1PG UK; 2 Public Health and Primary Care Directorate, Imperial College Healthcare NHS Trust, London, UK; 3 Department of Biostatistics and Health Informatics, Institute of Psychiatry, Psychology and Neuroscience, King’s College London, De Crespigny Park, London, UK; 4 Centre for Implementation Science, Health Services and Population Research Department, Institute of Psychiatry, Psychology and Neuroscience, King’s College London, De Crespigny Park, London, UK; 5 South West Sydney Clinical School, UNSW Australia, Sydney, NSW Australia; 6 Ingham Institute for Applied Medical Research, Liverpool, NSW Australia; 7 Woolcock Institute of Medical Research, University of Sydney, Sydney, NSW Australia; 8 Centenary Institute of Cancer Medicine & Cell Biology, University of Sydney, Sydney, NSW Australia; 9 School of Public Health and Menzies Centre for Health Policy, University of Sydney, Sydney, NSW Australia; 10 Nutrition Policy Institute, University of California, College of Agriculture and Natural Resources, Berkeley, California USA; 11 Sydney Local Health District, Sydney, NSW Australia; 12 Department of Respiratory Medicine, Liverpool Hospital, Liverpool, NSW Australia

**Keywords:** Allergic sensitisation, Air pollution, PUFAs, Fish oil, Lung function, Children, Birth cohort

## Abstract

**Background:**

Studies of potential adverse effects of traffic related air pollution (TRAP) on allergic disease have had mixed findings. Nutritional studies to examine whether fish oil supplementation may protect against development of allergic disease through their anti-inflammatory actions have also had mixed findings. Extremely few studies to date have considered whether air pollution and dietary factors such as fish oil intake may interact, which was the rationale for this study.

**Methods:**

We conducted a secondary analysis of the Childhood Asthma Prevention Study (CAPS) birth cohort, where children were randomised to fish oil supplementation or placebo from early life to age 5 years. We examined interactions between supplementation and TRAP (using weighted road density at place of residence as our measure of traffic related air pollution exposure) with allergic disease and lung function outcomes at age 5 and 8 years.

**Results:**

Outcome information was available on approximately 400 children (~ 70% of the original birth cohort). Statistically significant interactions between fish oil supplementation and TRAP were seen for house dust mite (HDM), inhalant and all-allergen skin prick tests (SPTs) and for HDM-specific interleukin-5 response at age 5. Adjusting for relevant confounders, relative risks (RRs) for positive HDM SPT were RR 1.74 (95% CI 1.22–2.48) per 100 m local road or 33.3 m of motorway within 50 m of the home for those randomised to the control group and 1.03 (0.76–1.41) for those randomised to receive the fish oil supplement. The risk differential was highest in an analysis restricted to those who did not change address between ages 5 and 8 years. In this sub-group, supplementation also protected against the effect of traffic exposure on pre-bronchodilator FEV_1_/FVC ratio.

**Conclusions:**

Results suggest that fish oil supplementation may protect against pro-allergic sensitisation effects of TRAP exposure. Strengths of this analysis are that supplementation was randomised and independent of TRAP exposure, however, findings need to be confirmed in a larger experimental study with the interaction investigated as a primary hypothesis, potentially also exploring epigenetic mechanisms. More generally, studies of adverse health effects of air pollution may benefit from considering potential effect modification by diet and other factors.

**Trial registration:**

Australia New Zealand Clinical Trial Registry. www.anzctr.org.au Registration: ACTRN12605000042640, Date: 26th July 2005. Retrospectively registered, trial commenced prior to registry availability.

**Electronic supplementary material:**

The online version of this article (10.1186/s12940-018-0370-5) contains supplementary material, which is available to authorized users.

## Background

A number of environmental health studies have found associations between ambient air pollution exposure and allergen sensitisation [1–3] although some studies have not, most notably a meta-analysis of five European cohorts in the European Study of Cohorts and Air Pollution Effects (ESCAPE) [4]. Meanwhile, nutritional studies have examined whether fish oil intake may protect against development of allergic disease through their known anti-inflammatory actions [5]. Previous randomized controlled trials of fish oil supplementation in pregnancy, lactation and from birth [6–13] have had mixed findings without consistently observed benefits of supplementation on either asthma or allergy development in childhood. Extremely few studies to date have considered whether dietary factors may modify impacts of air pollution, which could be a potential explanation for inconsistent findings in both air pollution and fish oil supplementation studies.

In this study we investigated the hypothesis that the risk of allergic sensitisation in relation to traffic related air pollution (TRAP) exposure in childhood would be modified (reduced) by fish oil supplementation. We conducted a secondary analysis of the Childhood Asthma Prevention Study (CAPS), set up as a randomised controlled trial investigating whether fish oil supplementation or house dust mite (HDM) exposure reduction implemented from the first year of life to age 5 years reduced the risk of development of asthma or atopy in children at high risk of developing these due to a family history of asthma [14]. No effect of either intervention was demonstrated at either 5 [15] or 8 [7] years. We have demonstrated, in a subsequent cross-sectional analysis of the whole cohort participating at age 8 years [16], that children living in homes with higher weighted road density, as a marker of TRAP, had higher probabilities of allergic sensitisation to house dust mite and allergic rhinitis and also showed small decrements in mid-expiratory flows.

## Methods

### Randomisation in the original RCT

Six hundred sixteen children born in 1997–2000 were included in the original RCT. Recruitment took place before birth – pregnant women whose child would be at high risk of developing asthma, because of a parent or a sibling with a current diagnosis of asthma or with frequent wheeze, were recruited from antenatal clinics of six hospitals in Sydney, Australia (see Additional file 1). In the ‘active supplement intervention’ group, children were given capsules of 500 mg of tuna fish oil added to infant formula from birth, or if breast fed, from age 6 months along with monounsaturated cooking oils and fat spreads, which were commenced when the child started solid foods and were continued to the age of 5 years. The tuna fish oil contained 37% omega-3 polyunsaturated and 6% omega-6 fatty polyunsaturated acids, 24% monounsaturated acids, 28% saturated fatty acids and 5% minor fatty acids. The control group were supplied with oils and margarines designed to maintain the low omega-3 and high omega-6 intake seen in the Australian population, as detailed elsewhere [14, 15]. Compliance with supplementation was checked regularly and plasma levels of omega-3 were found to be significantly higher in the active supplement intervention group and plasma omega-6 levels significantly lower compared with controls at age 18 month, 3 year and 5 year clinical follow-ups [15].

### Weighted road density exposure assignment

Weighted road density (WRD) at place of residence was used as an indicator of exposure to TRAP, using a method designed to predict air pollution for areas where air quality monitoring and traffic count data were not available [17]. Address was only retained electronically from age 8 years onwards but not from birth. Paper records were hand searched to identify addresses from ages 5 to to 8 years for 129 participants who indicated at age 8 years that they had moved since the age 5 year follow-up. Timing of moving house could not be reliably inferred and address records for ages < 5 years were incomplete so it was not possible to construct a lifetime residential history. Addresses were geocoded using the Geographic National Address File (GNAF) with additional investigation by hand using Google Maps; 99.4% of addresses available were successfully geocoded.

Each study subject still residing in New South Wales was assigned a WRD score comprising the weighted sum of the lengths of road within 50 m radius of the property centroid of the main place of residence at the time of the clinic visits at age 5 and 8 years. As described previously [17], motorways, arterial roads and primary roads were given a weighting of 3, distributor roads a weighting of 2 and local roads given a weighting of 1. Radii of 50 m were chosen given that concentrations of nitrogen dioxide (NO_2_), often used as a marker of TRAP, have been shown to fall rapidly within that distance from roads [18] and because our previous work showed that WRD within 50 m of home, was associated with an increased probability of having positive HDM SPT and HDM specific IgE, with doctor-diagnosed allergic rhinitis and small decreases of pre-and post- bronchodilator mid-expiratory flow measures [16].

### Outcome assessment

Questionnaires were administered by nurses and obtained information on symptoms, diagnosed asthma and allergic rhinitis, and various environmental factors and confounders. Clinical assessment at both age 5 and 8 years included height, weight, allergen skin prick testing (SPT, classified positive if weal ≥3 mm at 10 min), blood samples for total IgE, interleukin (IL-)5 and IL-10 in vitro T cell cytokine responses to HDM extract (responders ≥10 pg/ml), and lung function (Forced Expiratory Volume in one second (FEV_1_), Forced Vital Capacity (FVC), Forced Expiratory Flow at 50% of FVC (FEF_50_), Forced Expiratory Flow at 25–75% of FVC (FEF_25–75_), Peak Expiratory Flow (PEF)). Spirometric lung function was measured pre- and post- administration of bronchodilator (salbutamol 200 μg). At the age 8 years assessment, methacholine challenge test was performed in all consenting children with baseline FEV_1_ > 70% predicted. The measurement of bronchodilator response was conducted on a different day from all other clinical measurements. (For further details please see Additional file 1).

### Ethics

Informed written consent was given by the parents of participating children and the study was approved by the Human Research Ethics Committees of the University of Sydney #12–2004/7954, Children’s Hospital at Westmead HREC96/7/4.17(154), Sydney South West Area Health Services #96/80 and Sydney Local Health District #08/RPAH/472.

### Statistical analysis

Associations between WRD at place of residence and binary allergic and respiratory outcomes at age 5 and age 8 years (SPTs, asthma, wheeze, allergic rhinitis, eczema, and interleukins) were investigated using a random intercept Poisson model with robust error variance [16, 19]. A random intercept linear regression model was used to analyse the effect of WRD on lung function (FEV_1_, FVC and FEV_1_/FVC ratio) and total IgE. Lung function analyses were conducted on log-transformed variables and included (a priori defined) covariates of age at testing, weight and height. Effect modification of the association of the above allergic and respiratory outcomes with randomisation to fish oil supplementation (yes/no) was assessed by the inclusion of an interaction term in the random intercept Poisson and linear models. Relative Risks (RRs) provided are expressed as increase in risk per unit increase in weighted road density, representing 100 m local road or 33.3 m of motorway within 50 m of the home. Associations between age 5 to age 8 years were not statistically significantly different from each other (*p* values of interaction terms between fish oil supplementation, weighted road density and time (age 5 and age 8) for questionnaire and clinical outcomes > 0.05, data not shown). Therefore, we conducted a repeated measures analysis using exposure and outcome observations from age 5 and age 8 years to increase statistical power. We also restricted analyses to those who had not moved house between age 5 and 8 years who might be expected to have less exposure misclassification for exposure to TRAP.

Further sensitivity analyses were conducted stratified by atopy (any positive SPT at age 8 years) because our previous analyses not taking account of randomisation suggested atopic children might be more sensitive to TRAP [16].

All analyses adjusted for the following potential confounders identified a priori: sex, ethnicity, environmental tobacco exposure during pregnancy and childhood, breast-feeding to age 6 months, current or previous dog or cat ownership, gas cooking, parental education. Analyses were performed using STATA 13.1.

## Results

**Fig. 1:**
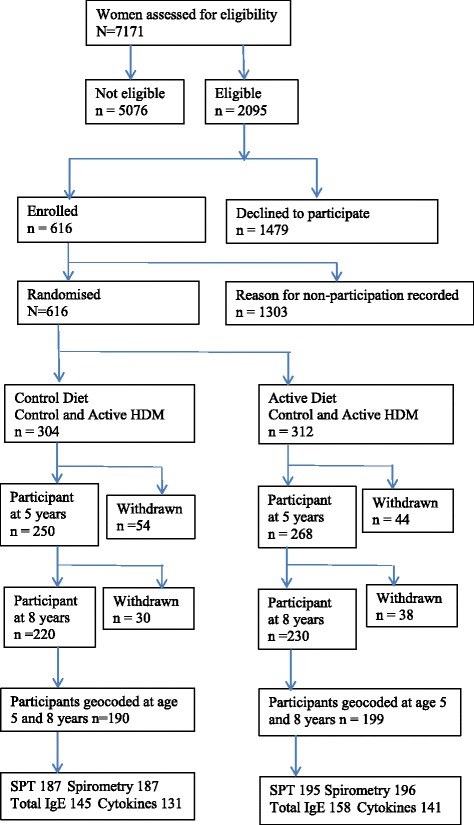
Flow-chart of CAPS study participants with respect to original randomisation

There were 616 children in the original birth cohort and 560 remained living in New South Wales with an address at age 8 years that could be geocoded. At age 5 and age 8 years respectively there were 418 (74% of 560) and 419 (75%) children with questionnaire information on current asthma symptoms; 409 (73%) and 382 (68% of 560) with SPTs; and 382 (68%) and 410 (73%) with lung function tests. At age 8 years, 121 (28.9%) of the 419 children with questionnaire information and 104 (27.2%) of the 382 children with SPTs had moved house since age 5 years. A flow chart of the sample selection is shown in Fig. 1.

Characteristics of the children at age 5 and age 8 are presented in Table 1. At age 5 years, HDM was the most common allergen children were sensitised to: 29% had positive SPT. The next most common allergens were grasses (11% with positive SPTs). Sensitisation was much lower (< 10%) for ingested (food) allergens than inhalant allergens (see Table 1 for definitions). At age 5 years, 31% had wheezed in the last 12 months and 27% had ever had doctor diagnosed asthma. Respective percentages for age 8 years were HDM SPT 36%, wheeze in last 12 months 27%, and ever doctor diagnosed asthma 41%. Half (51%) of the children had been randomised to fish oil supplementation. Analysis of plasma fatty acids for omega3/6 ratio at the clinic visit showed slightly higher mean ratios in those in the supplementation group at age 5 but not at age 8 years. Distribution of confounders is shown in Additional file 2: Table S1.

**Table 1:** Allergic sensitisation, self-reported allergic disease, lung function testing, weighted road density and potential confounders at age 5 and 8 years

	At age 5 years		At age 8 years	
Positive skin prick tests (Skin prick test > 3 mm)	Number (%)	n	Number (%)	n
Any of 11 inhalant and food allergens^a^	151 (36.9%)	409	173 (45.3%)	382
Inhalant allergen^b^	145 (35.4%)	409	170 (44.5%)	382
Ingested allergen^c^	25 (6.1%)	409	30 (7.9%)	382
House dust mite (HDM)	120 (29.3%)	409	137 (35.9%)	382
Ryegrass	43 (10.5%)	409	69 (18.1%)	381
Grass mix	43 (10.5%)	409	51 (13.4%)	380
*Alternaria tenuis*	29 (7.1%)	409	43 (9.7%)	380
Cockroach	18 (4.4%)	409	18 (4.7%)	381
Cat dander	16 (4.5%)	409	23 (4.5%)	382
Cytokines and total IgE				
HDM specific IL-5 responder	78 (25.4%)	307	78 (30.23%)	258
HDM specific IL-10 responder	150 (51.1%)	293	121 (46.7%)	281
Total IgE (kU/L)	Median:70 IQR(25–186)	341	Median:118 IQR(37–392)	303
Questionnaire variables				
Doctor-diagnosed asthma	114 (27.2%)	418	119 (28.40%)	419
Ever Doctor-diagnosed Asthma	141 (33.6%)	419	170 (40.5%)	419
Wheeze in last 12 months	130 (31.1%)	418	114 (27.2%)	419
Wheeze in the last 12 months + asthma diagnosis	83 (19.8%)	418	91 (21.7%)	419
Cough in last 12 months at least four times	143 (34.2%)	418	101 (24.1%)	419
Doctor diagnosed allergic rhinitis	23 (5.5%)	418	52 (12.4%)	419
Doctor diagnosed eczema	119 (28.47%)	418	129 (30.7%)	419
Eczema in the last 12 months	92 (22.1%)	416	58 (14.3%)	405
Spirometry	Mean (SD)	N	Mean (SD)	N
FEV_1_ pre bronchodilator % predicted	115.8% (17.7)	328	100.1% (11.8)	396
FVC pre bronchodilator % predicted	124.3% (20.3)	323	98.9% (11.6)	390
FEV_1_ post bronchodilator % predicted	120.0% (18.4)	323	105.9% (11.1)	392
FVC post bronchodilator % predicted	128.2% (19.7)	323	99.5% (10.6)	386
FEV_1_/FVC ratio pre bronchodilator	0.93 (0.06)	328	0.89 (0.07)	391
FEV_1_/FVC ratio post bronchodilator	0.93 (0.06)	328	0.93 (0.05)	387
pre Peak Expiratory Flow (PEF)	2.28 (0.47)	326	203 (37.1)	410
post Peak Expiratory Flow (PEF)	2.33 (0.53)	321	211 (38.2)	406
Pre forced expiratory flow at 50% vital capacity (FEF_50_)	1.60 (0.41)	326	2.18 (0.55)	410
Post forced expiratory flow at 50% vital capacity(FEF_50_)	1.77 (0.51)	321	2.51 (0.53)	406
Pre forced expiratory flow at mid-expiratory phase(FEF_25–75_)	1.46 (0.37)	326	2.11(0.43)	410
Post forced expiratory flow at mid-expiratory phase(FEF_25–75_)	1.61 (0.46)	321	2.35 (0.43)	406
Exposure	Mean (SD)	N	Mean (SD)	N
Weighted road density within 50 m of home, where one unit represents 100 m local road or 33.3 m of motorway	1.04 (0.6)	390	1.1 (0.6)	389
Mean Omega3:Omega6 ratio (fish oil supplemented vs. non-supplemented ^(*p*-value significance)^)	0.18 vs. 0.14^(^***^)^	367	0.15 vs. 0.15^(NS)^	314
	Number (%)	N	Number (%)	N
Fish oil supplementation at age 5 years	214 (51%)	419	214 (51%)	419
Urban environment	392 (93.7%)	418	421 (91.9%)	458

^a^Any of egg white, egg yolk, salmon, tuna, peanuts, *D. Pteronyssinus*, cat dander, cockroach, alternaria, rye grass, grass mix, dog hair, aspergillus

^b^Any of *D. Pteronyssinus*, cat dander, cockroach, rye grass, grass mix, alternaria, dog hair, aspergillus

^c^Any of egg white, egg yolk, salmon, tuna, peanuts

***t-test *p*-value < 0.001

^NS^t-test *p*-value > 0.1

### Analysis for age 5 years and for age 8 years

Interactions between fish oil supplementation and WRD were suggested for HDM, inhalant and all-allergen SPTs and for HDM-specific IL-5 (Table 2) at age 5 years, but not for other outcomes or for lung function tests (Tables 2 & 3). Relative risks of positive skin prick test to HDM were RR 1.74 (95% CI 1.22–2.48) per 100 m local road or 33.3 m of motorway within 50 m of home for those randomised to the control group and 1.03 (0.76–1.41) for those randomised to the active supplement intervention. For HDM-specific IL-5 the RR was 1.83 (1.21–2.76) for the control group and 0.653 (0.38–1.11) in the active supplement intervention group. Results for age 8 years showed statistical significance for the interaction term for both IL-5 and IL-10 (Additional file 2: Tables S2 and S3), but with lower RRs (IL-5 RR 1.52 (1.16–2.00)).

**Table 2:** Associations of selected allergic and respiratory outcomes at age 5 years in relation to weighted road density within a 50 m radius of home adjusted for potential confounders stratified by fish oil supplementation

Total IgE	No fish oil supplementation				Fish oil supplementation				*p*-value for interaction
	N	Mean difference	95% CI	*p*-value	N	Mean difference	95% CI	*p*-value	
Total IgE (kU/L)	165	−0.023	−0.29−0.34	0.760	161	−0.18	−0.34−0.20	0.785	0.291
Questionnaire variables		RR				RR			
Doctor diagnosed asthma	186	0.91	0.58–1.43	0.689	186	1.18	0.82–1.71	0.370	0.384
Ever had doctor diagnosed asthma	186	0.91	0.63–1.29	0.556	186	1.10	0.84–1.44	0.746	0.423
Wheeze in the last 12 months	186	1.18	0.81–1.72	0.387	186	1.05	0.79–1.39	0.724	0.664
Wheeze in the last 12 months & asthma	186	1.08	0.60–1.95	0.798	186	0.90	0.59–1.38	0.641	0.770
Cough more than 4 times in the last 12 months	186	1.14	0.81–1.59	0.459	186	1.20	0.95–1.53	0.132	0.794
Doctor diagnosed Eczema	186	1.13	0.78–1.65	0.514	186	1.43	1.10–1.87	0.007	0.631
Eczema in the last 12 months	185	1.46	1.01–2.11	0.043	186	1.34	0.96–1.87	0.085	0.882
Doctor diagnosed allergic rhinitis	186	1.27	0.61–2.64	0.528	186	3.44	1.82–6.47	0.000	0.969
Skin prick tests									
Any of 11 inhalant and food allergens	181	**1.34**	**1.00–1.80**	**0.054**	**186**	**0.90**	**0.67–1.21**	**0.493**	**0.042**
Inhalant allergen	181	**1.40**	**1.04–1.89**	**0.029**	**186**	**0.91**	**0.67–1.23**	**0.544**	**0.027**
Ingested allergen	181	0.51	0.10–2.63	0.424	186	1.28	0.55–2.99	0.571	0.695
House dust mite (HDM)	181	**1.74**	**1.22–2.48**	**0.002**	**186**	**1.03**	**0.76–1.41**	**0.840**	**0.015**
Alternaria tenuis	181	0.27	0.11–0.64	0.003	186	0.96	0.51–1.82	0.906	0.567
Grass mix	181	1.34	0.71–2.55	0.368	186	1.04	0.56–1.92	0.910	0.963
Rye Grass	181	1.33	0.70–2.53	0.382	185	1.08	0.66–1.78	0.761	0.906
HDM-specific cytokines									
IL-5 (> 10 pg/ml)	139	**1.83**	**1.21–2.76**	**0.004**	**139**	**0.65**	**0.38–1.11**	**0.117**	**0.003**
IL-10 (> 10 pg/ml)	131	1	0.83–1.22	0.969	133	0.98	0.83–1.16	0.817	0.981

All models are adjusted for sex, father’s education, mother’s education, environmental tobacco smoke exposure, breastfed to 6 months, any dog owned by 5 or 8 years, any cat owned by 5 or 8 years, maternal smoking in pregnancy, gas cooking at home

Interaction term of fish oil supplementation with weighted road density was added as a separate term into a Poisson model

Mean difference, Relative Risks (RRs) and 95% confidence intervals (95% CI) represent increase in risk per unit increase in weighted road density, representing 100 m local road or 33.3 m of motorway within 50 m of the home. N = number of children

**Table 3:** Associations of lung function measurements at age 5 years in relation to weighted road density within a 50 m radius of home adjusted for potential confounders stratified by fish oil supplementation

	No fish oil supplementation				Fish oil supplementation				*p*-value for interaction
	N	Mean Difference	95% CI	*p*-value	N	Mean Difference	95% CI	*p*-value	
log (FEV1 pre bronchodilator (L))	154	−0.04	−0.09−0.01	0.083	147	−0.04	−0.08−0.01	0.100	0.760
log (FEV1 post bronchodilator (L))	151	− 0.03	− 0.08−0.01	0.150	146	−0.03	− 0.08−0.02	0.188	0.808
log (FVC pre bronchodilator (L))	154	−0.05	−0.10−0.01	0.102	147	−0.03	−0.08−0.01	0.171	0.854
log (FVC post bronchodilator (L))	151	−0.03	−0.08−0.03	0.306	146	−0.01	−0.06−0.04	0.641	0.950
FEV1/FVC ratio pre bronchodilator	154	**−0.05**	**−0.10−0.00**	**0.037**	147	−0.03	−0.07−0.01	0.126	0.932
FEV1/FVC ratio post bronchodilator	151	−0.04	−0.08−0.01	0.102	146	−0.03	−0.08−0.01	0.175	0.908
pre Peak Expiratory Flow (PEF)	154	−0.05	−0.10−0.00	0.073	147	−0.03	−0.07−0.02	0.237	0.903
post Peak Expiratory Flow (PEF)	151	−0.03	−0.07−0.02	0.240	146	−0.01	−0.05−0.03	0.727	0.795
Pre forced expiratory flow at 50% vital capacity (FEF_50_)	154	0	−0.02−0.01	0.623	147	−0.01	−0.02−0.01	0.549	0.935
Post forced expiratory flow at 50% vital capacity (FEF_50_)	151	−0.01	−0.02−0.01	0.349	146	**−0.02**	**−0.04−0.00**	**0.022**	0.408
Pre forced expiratory flow at mid-expiratory phase (FEF_25–75_)	102	5.30	−6.24−16.85	0.364	99	−0.32	−14.53−13.88	0.964	0.386
Post forced expiratory flow at mid-expiratory phase (FEF_25–75_)	100	6.23	−6.04−18.50	0.316	98	−0.49	−14.22−13.24	0.944	0.410

All models are adjusted for age at spirometry, height at spirometry, weight at spirometry, sex, father’s education, mother’s education, environmental tobacco smoke exposure, breastfed to 6 months, any dog owned by 5 or 8 years, any cat owned by 5 or 8 years, maternal smoking in pregnancy, gas cooking at home

Interaction term of fish oil supplementation with weighted road density was added as a separate term into a random intercept Poisson model. Mean difference, Relative Risks (RRs) and 95% confidence intervals (95% CI) represent increase in risk per unit increase in weighted road density, representing 100 m local road or 33.3 m of motorway within 50 m of the home. N = number of children

### Repeated measures analysis for age 5 and 8 years

The repeated measures analysis that combined outcomes measured at ages 5 and age 8 years was suggestive of interactions for HDM-specific IL-5 (Additional file 2: Tables S4 and S5); again no effect was seen on lung function. When restricting the repeated measures analysis to those who did not move house between the age of 5 and 8 years, interactions between fish oil supplementation and weighted road density for positive SPT for HDM, inhalant and all-allergen SPTs and for HDM-specific IL-5 responses (Table 4) showed a similar pattern to that seen for age 5 years (Table 2) but with higher RRs. Specifically, for WRD and HDM SPT, the RR was 2.36 (1.34–4.14) for the control group and 0.97 (0.67–1.42) for the ‘active supplement intervention’ group. Results for lung function (Table 5) suggested interaction for pre- but not post-bronchodilator FEV_1_/FVC ratio, with a 3% (5% to 1%) decrease in FEV_1_/FVC ratio for the control group but no effect among children in the ‘active supplement intervention’ group. Restricting analyses to those who were atopic did not suggest significant interaction for the supplement intervention (Additional file 2: Tables S6 and S7), but numbers in each cell were small.

**Table 4:** Repeated measures analysis of combined age 5 and 8 years: Associations of selected allergic and respiratory outcomes in relation to weighted road density within a 50 m radius of home stratified by fish oil supplementation and restricted to non-movers between age 5 and 8 years

Total IgE	No fish oil supplementation				Fish oil supplementation				*p*-value for interaction
	N	Mean difference	95% CI	*p*-value	N	Mean difference	95% CI	*p*-value	
Total IgE (kU/L)	95	−0.023	−0.28–0.24	0.860	95	−0.11	−0.36–0.15	0.414	0.786
Questionnaire variables		RR				RR			
Doctor diagnosed asthma	126	1.11	0.66–1.89	0.688	111	0.94	0.64–1.40	0.781	0.763
Ever had doctor diagnosed asthma	126	1.03	0.66–1.62	0.889	111	0.78	0.54–1.16	0.231	0.387
Wheeze in the last 12 months	126	1.17	0.74–1.87	0.499	111	0.87	0.64–1.21	0.433	0.472
Wheeze in the last 12 months & asthma	126	1.23	0.65–2.36	0.525	111	0.71	0.46–1.13	0.155	0.308
Cough more than 4 times in the last 12 months	126	1.26	0.83–1.90	0.280	111	1.19	0.85–1.67	0.308	0.955
Doctor diagnosed Eczema	126	1.33	0.85–2.06	0.209	111	1.25	0.85–1.83	0.260	0.749
Eczema in the last 12 months	125	1.57	0.88–2.78	0.123	110	1.01	0.58–1.77	0.973	0.467
Doctor diagnosed allergic rhinitis	126	2.02	0.90–4.51	0.086	111	**2.38**	**1.01–5.62**	**0.048**	0.606
Positive Skin prick tests									
Any of 11 inhalant and food allergens	**121**	**1.62**	**1.08–2.45**	**0.021**	107	0.75	0.53–1.09	0.131	**0.031**
Inhalant allergen	**121**	**1.69**	**1.10–2.60**	**0.016**	107	0.77	0.54–1.11	0.165	**0.031**
Ingested allergen	121	1.49	0.45–4.89	0.510	107	1.00	0.53–1.90	0.996	0.800
House dust mite (HDM)	**121**	**2.36**	**1.34–4.14**	**0.002**	107	0.97	0.67–1.42	0.902	**0.026**
Alternaria tenuis	121	0.88	0.37–2.05	0.762	107	0.71	0.38–1.35	0.299	0.500
Grass mix	121	1.57	0.63–3.92	0.334	107	0.81	0.43–1.55	0.535	0.406
Rye Grass	121	1.73	0.78–3.86	0.180	107	0.75	0.35–1.63	0.475	0.206
HDM sensitization									
IL-5 (> 10 pg/ml)	**83**	**2.05**	**1.26–3.32**	**0.003**	**79**	**0.61**	**0.40–0.92**	**0.0186**	**<0.001**
IL-10 (> 10 pg/ml)	82	1	0.76–1.31	0.996	77	0.87	0.72–1.03	0.112	0.672

All models are adjusted for sex, father’s education, mother’s education, environmental tobacco smoke exposure, breastfed to 6 months, any dog owned by 5 or 8 years, any cat owned by 5 or 8 years, maternal smoking in pregnancy, gas cooking at home

Interaction term of fish oil supplementation with weighted road density was added as a separate term into a random intercept Poisson model. Mean difference, Relative Risks (RRs) and 95% confidence intervals (95% CI) represent increase in risk per unit increase in weighted road density, representing 100 m local road or 33.3 m of motorway within given radius of the home. N = number of children

**Table 5:** Repeated measures analysis of combined age 5 and 8 years: Associations of lung function measurements in relation to weighted road density within a 50 m radius of home and stratified by fish oil supplementation restricted to non-movers between age 5 and 8 years

	No fish oil supplementation				Fish oil supplementation				*p*-value for interaction
	N	Mean Difference	95% CI	*p*-value	N	Mean Difference	95% CI	*p*-value	
log(FEV1 pre bronchodilator (L))	114	−0.06	−0.11−0.02	0.009	101	0	− 0.04−0.04	0.938	0.088
log(FEV1 post bronchodilator (L))	111	−0.04	−0.08−0.00	0.050	100	−0.01	− 0.05−0.02	0.505	0.508
log (FVC pre bronchodilator (L))	113	−0.03	−0.07−0.01	0.200	99	0	−0.04−0.04	0.997	0.680
log (FVC post bronchodilator (L))	110	−0.01	−0.05−0.03	0.587	98	0	−0.04−0.03	0.846	0.843
FEV1/FVC ratio pre bronchodilator	113	−0.03	−0.05--0.01	0.014	99	0	−0.02−0.02	0.979	0.031
FEV1/FVC ratio post bronchodilator	110	−0.02	−0.04−0.01	0.010	98	−0.01	−0.02−0.01	0.239	0.139
pre Peak Expiratory Flow (PEF)	91	4.33	−4.09−12.75	0.313	85	−0.55	−7.14−6.04	0.871	0.252
post Peak Expiratory Flow (PEF)	90	8.46	−0.07−16.98	0.051	84	−2.57	−9.29−4.14	0.453	0.056
Pre forced expiratory flow at 50% vital capacity (FEF_50_)	91	−0.09	−0.22−0.05	0.227	85	0.01	−0.11−0.13	0.893	0.380
Post forced expiratory flow at 50% vital capacity (FEF_50_)	90	−0.08	−0.23−0.08	0.333	84	−0.09	−0.22−0.04	0.19	0.757
Pre forced expiratory flow at mid-expiratory phase (FEF_25–75_)	91	−0.07	−0.18−0.05	0.270	85	−0.02	−0.12−0.08	0.743	0.747
Post forced expiratory flow at mid-expiratory phase (FEF_25–75_)	90	−0.03	−0.16−0.11	0.701	84	−0.08	−0.20−0.03	0.161	0.369

All models are adjusted for age at spirometry, height at spirometry, weight at spirometry, sex, father’s education, mother’s education, environmental tobacco smoke exposure, breastfed to 6 months, any dog owned by 5 or 8 years, any cat owned by 5 or 8 years, maternal smoking in pregnancy, gas cooking at home

Interaction term of fish oil supplementation with weighted road density was added as a separate term into a random intercept Poisson model. Mean difference, Relative Risks (RRs) and 95% confidence intervals (95% CI) represent increase in risk per unit increase in weighted road density, representing 100 m local road or 33.3 m of motorway within 50 m of the home. N = number of children

## Discussion

To examine our hypothesis that fish oil supplementation might modify adverse effects of TRAP, we conducted a secondary analysis of a RCT that had previously shown no effect overall of fish oil supplementation on incidence of asthma or allergic sensitisation [7]. Analyses suggested protective effect modification by randomisation group status on associations between WRD within 50 m of home and HDM sensitisation whether assessed by SPT or HDM-specific cytokine responses and on pre- (but not post-) bronchodilator FEV_1_/FVC ratio. Impacts of the active supplement intervention, which included fish oil supplementation and provision of canola-based oils and spreads, were also seen for all-allergen and inhalant allergen SPTs although these aggregate outcomes were strongly influenced by HDM-specific sensitisation status as this was the most common allergen to which participants were sensitised (Table 1).

Previously [16], we showed in this cohort of children with a family history of asthma or atopy that WRD within 50 m of home, an index of exposure to TRAP, was associated with the higher probability of having positive HDM SPT and HDM specific IgE, for doctor-diagnosed allergic rhinitis and small decreases of pre-and post- bronchodilator PEF, FEF_50_ and FEF_25–75_ [16], but we did not see associations with HDM-specific cytokine responses.

The aim of the fish oil supplementation was to alter the balance of n-6 and n-3 polyunsaturated fatty acids (PUFA), thus reducing the production of eicosanoid mediators produced from the n-6 PUFA arachidonic acid [20] that are key mediators of the airway inflammatory response. There may have been additional impacts on leukocyte chemotaxis, adhesion molecule expression and leukocyte-endothelial adhesive interactions, production of inflammatory cytokines and T-helper 1 lymphocyte activity [20], and reduction of inflammatory mediators [21].

It is difficult to conduct randomization of fish consumption, but observational studies of intake of fish in childhood have reported protective effects on asthma (four studies) [11] and allergic rhinitis [22]. Such studies have generally considered fish and fish oil intake in isolation and have not investigated co-factors or environmental agents that may have a role in the development of allergic disease.

The novel feature of this study is the assessment of environment-environment interactions that may, at least partly, explain inconsistencies in previous research that ignored these interactions. An RCT in children aged 6 years in South Africa found that long chain fish oil supplementation prevented increases in (primarily respiratory) infection associated with iron supplementation [23], which the authors attributed to iron-induced oxidative stress and inflammation. This is relevant here as TRAP produces inflammation that may in part relate to oxidative potential of metals included in the particulate fraction [18]. As well as potential for fish oils to reduce inflammatory responses [5, 20], other mechanisms by which fish oils may modify effects of TRAP include indirect influence on immune responses, via potential to influence the composition of the microbiome [24, 25].

Epigenetics may help provide a mechanism explaining the findings from this study. Traffic related air pollution has been shown to induce changes in methylation levels in genes relevant to asthma and allergic sensitisation [26, 27] and histone H3 modification [28], probably by increasing oxidative stress and pro-inflammatory responses. Epigenetic variations near and through the fatty acid desaturase (*FADS*) gene cluster account for variations in circulating and cellular long-chain PUFAs, the bioactive metabolites synthesised from dietary PUFAs [29]. Supplementation with dietary n-3 PUFA has been shown to be associated with DNA methylation of PUFA biosynthesis genes [30], leading to gene silencing of inflammatory pathways [31, 32]. In this study, the fatty acid dietary supplementation occurred during the first 5 years of life which coincides with a critical time in development of the immune system, therefore a hypothesis from our findings is that epigenetic modification induced by exposure to traffic related air pollution was protected against by epigenetic changes in children who received the dietary intervention.

Particular strengths of this study are that we conducted a post hoc analysis based on randomized allocation to a fish oil intervention. Compliance with the fish oil intervention was good in assessments conducted at 18 months, 3 and 5 years, verified by plasma omega-3/omega-6 ratios [15]. We did not conduct analyses based on ratios as these represent a snapshot at the time of clinic visit and may not be a good marker of long-term use of the supplement. Findings suggest it may be possible to modify sensitisation within the first 5 years of life, but, as the interventions were from birth until age 5 years, it was not possible to investigate optimal time windows for these effects within this time period. We conducted repeated measures analyses combining exposure and outcome observations from age 5 and age 8 years as there was no statistically significant difference between observed association patterns for outcomes at age 5 and age 8 years – the lack of difference suggests there was no important change in the protective effect of the supplement in the 3 year period following cessation of the active intervention.

Experimental studies have shown that diesel particulates act as adjuvants and increase sensitization to new allergens [33]. We used WRD at the place of residence as an indicator of exposure to TRAP. We did not have information on air pollutant concentrations or traffic count in the street of residence, but a previous evaluation of the WRD measure found this to be as strongly predictive of NO_2_ (measured by passive samplers) as was traffic volumes in a previous study in 2006–7 involving 38 monitoring sites in Sydney [17].

We found interactions between fish oil supplementation and HDM sensitization, and for pre- but not post-bronchodilator FEV1/FVC ratio (in non-movers, i.e. reducing exposure misclassification bias), but not for symptoms. One of the issues with studying asthma symptoms (breathless, cough and wheeze) is that they may be caused by a heterogeneous range of conditions with different causes. For example, up to six distinct wheezing phenotypes have been suggested [34, 35]. Furthermore, symptoms are substantially affected by the use of medications. Hence, it is not particularly surprising the effect of one specific cause for symptoms, as tested here, might be difficult to detect. In contrast to symptoms, allergic sensitisation is a less heterogeneous outcome and is not influenced by treatment.

Our TRAP proxy measure, WRD within 50 m of each residence, was constructed and evaluated at the time children were aged 8 years. We were unable to retrospectively construct a lifetime residential history and as a result we were unable to look at the effect of the timing of TRAP exposure on the observed associations. We applied the WRD measure to place of residence at age 5 years (as TRAP exposures were expected to be similar over the 3 year period) and age 8 years, but this will have led to some exposure misclassification. We note that the effects were greater in the analyses that were restricted to non-movers between age 5 and age 8 years. This is consistent with the expected impact of a reduction in exposure misclassification.

This was a post hoc analysis of a relatively small cohort with potential selection bias related to both recruitment criteria and to selective drop-out of children. Just under a third of eligible pregnant mothers agreed to participate in the original RCT, however, this decision could not have been influenced by the child’s as yet unknown asthma or allergic status. A further quarter of children had dropped out of the cohort by age 8 years; this was not related to exposure (WRD), but children who dropped out were more likely to have parents without a university education [16]. The latter might introduce bias that would reduce the size of an observed effect, as children of lower socio-economic status may be more likely to have a diet with a lower omega-3/omega-6 ratio, and therefore benefit more from fish oil supplementation. Some of the associations may have been a result of chance, due to the number of analyses conducted. However, findings were consistent over various sensitivity analyses and between independent measures of HDM allergen sensitisation – SPT and cytokine responses. Further, the RCT was intentionally conducted in children expected to be at higher genetic likelihood of allergic sensitisation because of a family history of asthma, representing a group most likely to benefit from the intervention, but which may limit generalisability. Findings should be viewed as a hypothesis generating analysis and would need replication before inferring causality.

## Conclusions

This study, conducted in children who were randomized to fish oil supplementation or placebo from birth to age 5 years, suggested that fish oil supplementation in early childhood may mitigate excess risk of allergic sensitisation associated with higher exposure to TRAP. This important interaction needs confirmation in independent cohorts and randomized trials, but the findings highlight the importance of studying environment-environment interactions.

## Additional files

Additional file 1: Supplementary methods. (DOC 72 kb)

Additional file 2: Supplementary results. **Table S1.** Descriptive table of potential confounders at follow-ups at age 5 and 8 years. **Table S2.** Associations of selected allergic and respiratory outcomes at age 8 years in relation to traffic density within a 50 m radius of home adjusted for potential confounders and stratified by fish oil supplementation. **Table S3.** Associations of lung function outcomes at age 8 years in relation to traffic density within a 50 m radius of home adjusted for potential confounders and stratified by fish oil supplementation. **Table S4.** Repeated measures analysis of combined age 5 and 8 years: Associations of selected allergic and respiratory outcomes in relation to traffic density within a 50 m radius of home adjusted for potential confounders stratified by fish oil supplementation. **Table S5.** Repeated measures analysis of combined age 5 and 8 years: Associations of lung function measurements in relation to traffic density within a 50 m radius of home adjusted for potential confounders and stratified by fish oil supplementation. **Table S6.** Repeated measures analysis of combined age 5 and 8 years in atopic children: Associations of selected respiratory outcomes in relation to traffic density within a 50 m radius of home stratified by fish oil supplementation. **Table S7.** Repeated measures analysis of combined age 5 and 8 years in atopic children. Associations of lung function measurements in relation to traffic density within a 50 m radius of home adjusted for potential confounders and stratified by fish oil supplementation. (DOC 195 kb)

## Abbreviations

CAPS: Childhood Asthma Prevention Study
ESCAPE: European Study of Cohorts and Air Pollution Effects
FEF_25–75_: Forced Expiratory Flow at 25–75% of FVC
FEF_50_: Forced Expiratory Flow at 50% of FVC
FEV_1_: Forced Expiratory Volume in one second
FVC: Forced Vital Capacity
GNAF: Geographic National Address File
HDM: House Dust Mite
IL-10: Interleukin-10
IL-5: Interleukin-5
NO_2_: Nitrogen dioxide
PEF: Peak Expiratory Flow
PUFA: Polyunsaturated Fatty Acids
RR: Relative Risk
SPT: Skin Prick Test
TRAP: Traffic Related Air Pollution
WRD: Weighted Road Density
