# Supplementary material for: Childhood fish oil supplementation modifies associations between traffic related air pollution and allergic sensitisation
Source: Environ Health. 2018 Mar 27;17:27. doi: 10.1186/s12940-018-0370-5 (PMC5870687; doi:10.1186/s12940-018-0370-5)
Supplement: Supplementary file 1 — Supplementary methods. (DOC 72 kb) [file 12940_2018_370_MOESM1_ESM.doc]

## Supplementary Methods

### Recruitment

Pregnant women whose unborn children were at high risk of developing asthma because of a parent or a sibling with a current diagnosis of asthma or with frequent wheeze were recruited from antenatal clinics in six hospitals in Sydney, Australia. Over 7000 women were screened using a short questionnaire. Women who satisfied the selection criteria were given further information about the study**;** these were:

Inclusion criteria:

- At least one parent or sibling with symptoms of asthma as assessed by screening questionnaire
- Reasonable fluency in English
- Telephone at home
- Reside within 30 km from center of recruitment

Exclusion criteria:

- Pet cat at home
- Families on strict vegetarian diet
- Multiple births
- Babies born earlier than 36 weeks gestation

Of 2095 women eligible for the study who were invited to participate, 616 (29.4%) agreed to participate, representing 8.6% of all women screened. For more information, see Marks et al, 2006 (1).

### Geocoding of addresses

All CAPS historical addresses in NSW in the database as at 28/11/2013 (up to age 16 years) were included together with addresses from additional extensive investigation of 129 participants who had indicated on questionnaire that they moved house between 5 and 8 years of age. For these participants, information on all of their addresses was obtained by manually hand searching hard copies of their CAPS files. In total the final address dataset included 2386 addresses. This included multiple records containing the same geocoded address but different record dates, the reason for this being whenever the participant’s contact details are updated, all contact details are copied and dated to a new record, so if the only thing that changed was a mobile telephone number this will result in another address entry but with a new date (indicating the participant was still resident at this address at this contact detail update). 2297 (96.2%) addresses were able to be geocoded using the Geographic National Address File (GNAF) and satisfied the criteria of “exact” match (i.e.“exact” allocation and “exact” description of the address). A further 75 (3.1%) addresses were located by hand using Google Maps and coordinates allocated leaving 14 records (0.6%) that could not be geocoded. Weighted traffic density was calculated based on the successfully geocoded latitude and longitude coordinates.

### Assigning weighted road traffic exposure

We calculated the age of every child at each time-point that their address was given using their date of birth**.** If there were no addresses between age>6 and age <9 we used an address at age >9. We always used the address closest in proximity to the clinical assessment date.

Unfortunately, there was not enough information in paper records on where participants’ lived in the first five years of life to construct a residential history age 0-5 years. We therefore restricted our analyses to the age 5 years and age 8 years follow-up (at the end of the fish oil supplementation and three years following this. We were not able to construct a residential history for the 129 children who had moved between age 5 and age 8 years as the date of moving was not clearly specified in the dataset, but we conducted a sensitivity analysis restricting age 8 age analyses to those who had not moved between age 5 and age 8 years.

### Questionnaires

Questionnaires were administered by nurses and obtained information on symptoms, diagnosed asthma and various environmental factors. Information about smoking in pregnancy was obtained by interview soon after delivery of the child.

For analyses, “doctor-diagnosed asthma” was defined as a doctor diagnosis of asthma reported at that follow-up, “ever doctor-diagnosed asthma” was defined as a doctor diagnosis of asthma reported in at least one follow-up (at 18 months, 3, 5 or 8 years). Doctor diagnosed rhinitis was defined as a doctor diagnosis of allergic rhinitis at that follow-up. Eczema was defined as either (eczema on examination at age 8 years or a reported recurrent itchy rash) together with use of topical treatments for eczema in the last 12 months.

The questionnaire was developed from a number of sources. Questions were those that had been used during previous CAPS assessments to allow for consistency of definitions between follow-ups (2), with addition of validated questions from the International Study of Asthma and Allergy (ISAAC) 6-7 year old core questionnaire to allow for comparison between this cohort and other international cohorts (3), and questions that were consistent with the Third International Paediatric Consensus Statement on the Management of Childhood Asthma to allow classification of asthma to be consistent with the current consensus statement (4).

### Clinical data

Clinical assessment at age eight years included height, weight, allergen skin prick testing (SPT), blood samples for total and specific IgE and cytokine responses, lung function and bronchodilator reversibility (5).

Atopy was measured using SPT as previously described (2). Glycerol and histamine phosphate (10mg/ml) were used as negative and positive controls, respectively. Food allergens tested were salmon, peanut mix, egg white, egg yolk, and tuna, and inhalant allergens tested were house dust mite *Dermatophagoides pteronyssinus* (HDM), cockroach, cat pelt, dog hair, *Alternaria alternata (tenuis)*, *Aspergillus fumigatus*, rye grass and a grass mix (Hollister-Stier, Spokane WA). Weals were measured at 10 minutes as the mean of the longest diameter and its perpendicular; values were rounded down to the nearest 1mm. A positive test was one where the weal was ≥3mm and also greater than the negative control weal.

Lung function (Forced Expiratory Volume in one second (FEV1) and Forced Vital Capacity (FVC)) was measured using a Spirocard (QRS Diagnostic, LLC, Plymouth, MN, USA) spirometer linked to a laptop computer running SpiroScore+ (V2.6) software (Bird Healthcare, Melbourne, Australia). Children also had reversibility measured 10 minutes after the administration of salbutamol (200 µg) via a large volume spacer. The measurement of bronchodilator response was conducted on a different day to all other clinical measurements. Predicted lung function values were calculated using NHANES III equations (6).

Our spirometry protocol was consistent with ATS/ERS lung function testing criteria (7). Post-bronchodilator spirometry was performed by a single experienced research nurse trained in spirometry. One single spirometer was used for all measurements. The spirometer was calibrated each day and was re-checked mid-way through the day for an extended day of testing. Selection of best value was according to ATS guidelines criteria for useable and acceptable curves that was programmed into the spirometry software.

As described in Weber-Chrysochoou et al (8), IL-5 and IL-10 T cell cytokine responses to HDM extract were determined as follows. Blood was collected into lithium heparin-containing tubes at the time of the clinical assessments and stored at room temperature until it was processed. Six hours within blood collection peripheral blood mononuclear cells (PBMC) were separated by centrifugation on a Ficoll-Paque density gradient (Amersham Biosciences) and washed twice in phosphate buffered saline (PBS). The cells were resuspended at 10^6^viable cells/ml in serum-free culture medium (AIM-V medium,GIBCO Invitrogen Corp, California, USA). One ml of PBMC was distributed in 24 well plates with (i) phytohaemaglutinin (PHA,Sigma-Aldrich Co, St. Louis. MO, USA) at 10mg/ml, (ii) aqueous sonicated extract of HDM (CSL Bioscience, Melbourne, Australia) at 50 mg/ml, or (iii) medium alone, and incubated at 37°C with 5% CO_2_. The cell-free supernatants were collected after 48 hours and stored at -70 degrees C. The released cytokines were measured by ELISA, as previously described (9, 10), using the cytokine IL-5 and IL-10 kits: BD OptEIA Human Sets, BD Biosiences Pharmingen, catalogue numbers 555202 and 555157 respectively. The limits of detection of these assays were established as the mean of the lowest measurable calibration point for each assay. The cytokine response in PHA-stimulated culture was treated as the positive control and medium only culture as the negative control. Cytokine levels from the HDM cultures were adjusted for background by deducting either the cytokine level from the corresponding negative control culture or the detection limit, whichever was greater. Results for samples in which levels of all the cytokines in the PHA culture were less than 3 times the corresponding cytokine level in the negative control culture (or the detection limit for that cytokine, whichever was greater) were considered invalid for all cytokines for that sample. Cytokine concentrations of IL-5 and and IL-10 in the HDM stimulated PBMC were classed as non-responders (<10 pg/ml) and responders (>10 pg/ml).

### Reference list for supplementary methods

1. Marks GB, Mihrshahi S, Kemp AS, Tovey ER, Webb K, Almqvist C, et al. Prevention of asthma during the first 5 years of life: a randomized controlled trial. The Journal of allergy and clinical immunology. 2006;118(1):53-61.

2. Peat JK, Mihrshahi S, Kemp AS, Marks GB, Tovey ER, Webb K, et al. Three-year outcomes of dietary fatty acid modification and house dust mite reduction in the Childhood Asthma Prevention Study. JAllergy ClinImmunol. 2004;114(4):807-13.

3. Asher MI, Keil U, Anderson HR, Beasley R, Crane J, Martinez F, et al. International study of asthma and allergies in childhood (ISAAC): rationale and methods. European Respiratory Journal. 1995;8(3):483-91.

4. Warner JO, Naspitz CK, Cropp GJA. Third International Pediatric Consensus statement on the management of childhood asthma. Pediatric Pulmonology. 1998;25(1):1-17.

5. Toelle BG, Ng KK, Crisafulli D, Belousova EG, Almqvist C, Webb K, et al. Eight-year outcomes of the Childhood Asthma Prevention Study. The Journal of allergy and clinical immunology. 2010;126(2):388-9, 9.e1-3.

6. Hankinson J, Odencrantz J, Fedan K. Spirometric Reference Values from a Sample of the General U.S. Population. American journal of respiratory and critical care medicine. 1999;159(1):179-87.

7. Miller MR, Hankinson J, Brusasco V, Burgos F, Casaburi R, Coates A, et al. Standardisation of spirometry. European Respiratory Journal. 2005;26(2):319-38.

8. Weber-Chrysochoou C, Crisafulli D, Kemp AS, Britton WJ, Marks GB, Investigators C. Allergen-specific IL-5 responses in early childhood predict asthma at age eight. PloS one. 2014;9(5):e97995.

9. Weber-Chrysochoou C, Crisafulli D, Almqvist C, Li Q, Kemp AS, Britton WJ, et al. IL-5 T-cell responses to house dust mite are associated with the development of allergen-specific IgE responses and asthma in the first 5 years of life. The Journal of allergy and clinical immunology. 2007;120(2):286-92.

10. Marks GB, Zhou J, Yang HS, Joshi PA, Bishop GA, Britton WJ, et al. Cord blood mononuclear cell cytokine responses in relation to maternal house dust mite allergen exposure. Clinical & Experimental Allergy. 2002;32(3):355-60.
