# Supplementary material for: Childhood fish oil supplementation modifies associations between traffic related air pollution and allergic sensitisation
Source: Environ Health. 2018 Mar 27;17:27. doi: 10.1186/s12940-018-0370-5 (PMC5870687; doi:10.1186/s12940-018-0370-5)
Supplement: Supplementary file 2 — Supplementary results. Table S1. Descriptive table of potential confounders at follow-ups at age 5 and 8 years. Table S2. Associations of selected allergic and respiratory outcomes at age 8 years in relation to traffic density within a 50 m radius of home adjusted for potential confounders and stratified by fish oil supplementation. Table S3. Associations of lung function outcomes at age 8 years in relation to traffic density within a 50 m radius of home adjusted for potential confounders and stratified by fish oil supplementation. Table S4. Repeated measures analysis of combined age 5 and 8 years: Associations of selected allergic and respiratory outcomes in relation to traffic density within a 50 m radius of home adjusted for potential confounders stratified by fish oil supplementation. Table S5. Repeated measures analysis of combined age 5 and 8 years: Associations of lung function measurements in relation to traffic density within a 50 m radius of home adjusted for potential confounders and stratified by fish oil supplementation. Table S6. Repeated measures analysis of combined age 5 and 8 years in atopic children: Associations of selected respiratory outcomes in relation to traffic density within a 50 m radius of home stratified by fish oil supplementation. Table S7. Repeated measures analysis of combined age 5 and 8 years in atopic children. Associations of lung function measurements in relation to traffic density within a 50 m radius of home adjusted for potential confounders and stratified by fish oil supplementation. (DOC 195 kb) [file 12940_2018_370_MOESM2_ESM.doc]

## Supplementary results

**Table S1. Descriptive table of potential confounders at follow-ups at age five and eight years**

|  | 5 years old | | 8 years old | |
| --- | --- | --- | --- | --- |
| **Confounders** |  |  |  |  |
| Male | 216 (51.6%) | 418 | 217(51.7%) | 419 |
| Father's highest level of education completed |  |  |  |  |
| 1 | 198 (47.83%) | 414 | 198 (47.7%) | 415 |
| 2 | 77(18.60%) | 414 | 77(18.5%) | 415 |
| 3 | 124(29.95%) | 414 | 125(30.1%) | 415 |
| 4 | 15(3.62%) | 414 | 15(3.6%) | 415 |
| Mother's highest level of education completed |  |  |  |  |
| 1 | 206 (49.2%) | 418 | 207 (49.4%) | 419 |
| 2 | 74(17.7%) | 418 | 74(17.6%) | 419 |
| 3 | 124(29.6%) | 418 | 124(29.5%) | 419 |
| 4 | 14 (3.3%) | 418 | 14(3.3%) | 419 |
| Environmental tobacco exposure during pregnancy and childhood | 161 (38.5%) | 418 | 161 (38.5%) | 418 |
| Breast-feeding to age 6 months | 176 (42.1%) | 418 | 176(42.0%) | 419 |
| Current or previous dog ownership | 267 (63.8%) | 418 | 267(63.7%) | 419 |
| Current or previous cat ownership | 79(18.9%) | 418 | 79(18.8%) | 419 |
| Mother smoking during pregnancy | 194(23.2%) | 418 | 97(23.1%) | 419 |
| Exposed to gas heating / cooking | 201(50%) | 402 | 201 (49.8%) | 403 |

**Table S2. Associations of selected allergic and respiratory outcomes at age 8 years in relation to traffic density within a 50m radius of home adjusted for potential confounders and stratified by fish oil supplementation**

|  |  | **no fish oil supplementation** | | |  | **fish oil supplementation** | | |  |
| --- | --- | --- | --- | --- | --- | --- | --- | --- | --- |
|  | **N** | **Mean difference** | **95% CI** | **p-value** | **N** | **Mean difference** | **95% CI** | **p-value** | **p-value for interaction** |
| Total IgE ( kU/L | 128 | -0.041 | -0.28-0.29 | 0.630 | 127 | 0.006 | -0.34-0.51 | 0.996 | 0.55 |
| **Questionnaire variables** |  | **RR** |  |  |  | **RR** |  |  |  |
| Doctor diagnosed asthma | 197 | 1.22 | 0.88-1.69 | 0.23 | 201 | 1.38 | 0.34-5.60 | 0.65 | 0.88 |
| Ever had doctor diagnosed asthma | 197 | 1.08 | 0.83-1.42 | 0.57 | 201 | 1.44 | 0.59-3.50 | 0.43 | 0.7 |
| Wheeze in the last 12 months | 197 | 1.33 | 0.90-1.95 | 0.15 | 201 | **0.21** | **0.09-0.53** | **<0.01** | 0.55 |
| Wheeze& asthma in the last 12 months | 197 | 1.31 | 0.83-2.08 | 0.25 | 201 | 0.14 | 0.02-1.06 | 0.06 | 0.95 |
| Cough more than 4 times in the last 12 months | 197 | 1.28 | 0.92-1.79 | 0.15 | 201 | 0.29 | 0.04-2.21 | 0.23 | 0.41 |
| Doctor diagnosed Eczema | 197 | 1.32 | 0.97-1.80 | 0.08 | 201 | 0.80 | 0.23-2.83 | 0.73 | 0.43 |
| Eczema in the last 12 months | 194 | 1.60 | 0.97-2.63 | 0.07 | 198 | 0.60 | 0.83-3.83 | 0.54 | 0.38 |
| Doctor diagnosed rhinitis | 197 | 1.10 | 0.66-1.81 | 0.72 | 201 | 0.12 | 0.43-5.09 | 0.687 | 0.22 |
| **Positive Skin prick tests** |  |  |  |  |  |  |  |  |  |
| Any of 11 inhalant and food allergens† | 181 | **1.23** | **1.02-1.48** | **0.03** | 189 | 1.27 | 0.93-1.73 | 0.14 | 0.16 |
| Inhalant allergen ‡ | 181 | **1.45** | **1.17-1.80** | **<0.001** | 189 | 1.10 | 0.40-3.00 | 0.85 | 0.12 |
| Ingested allergen ‡‡ | 181 | 1.34 | 0.66-2.71 | 0.42 | 189 | 1.27 | 0.95-1.68 | 0.11 | 0.57 |
| House dust mite (HDM) | 181 | **1.24** | **1.03-1.50** | **0.02** | 189 | 1.83 | 0.79-4.25 | 0.16 | 0.21 |
| Alternaria tenuis | 181 | 1.32 | 0.77-2.26 | 0.31 | 188 | 1.31 | 0.53-3.28 | 0.56 | 0.08 |
| Grass mix | 181 | 1.75 | 0.90-3.38 | 0.10 | 189 | 0.75 | 0.16-3.53 | 0.71 | 0.06 |
| Rye Grass | 181 | 0.75 | 0.20-2.89 | 0.68 | 189 | 2.20 | 0.14-4.26 | 0.52 | 0.92 |
| **HDM sensitization** |  |  |  |  |  |  |  |  |  |
| IL-5 (>10 pg/ml) | 113 | **1.52** | **1.16-2.00** | **<0.001** | 138 | 1.10 | 0.20-6.14 | 0.91 | **0.03** |
| IL-10 (>10 pg/ml) | 131 | **1.40** | **1.2-2.1** | **0.05** | 141 | 1.13 | 0.10-7.14 | 0.57 | **0.02** |

All models are adjusted for sex, father’s education, mother’s education, environmental tobacco smoke exposure, breastfed to 6 months, any dog owned by 5 or 8 years, any cat owned by 5 or 8 years, maternal smoking in pregnancy, gas cooking at home

Interaction term of fish oil supplementation with traffic density was added as a separate term into a random intercept Poisson model.

Mean difference, Relative Risks (RRs) and 95 % confidence intervals (95% CI) represent increase in risk per unit increase in traffic density, representing 100m local road or 33.3m of motorway within 50m of home.

N=number of children.

†Any of egg white, egg yolk, salmon, tuna, peanuts, D. Pteronyssinus, cat dander, cockroach, alternaria, rye grass, grass mix, dog hair, aspergillus

‡ Any of D. Pteronyssinus, cat dander, cockroach, rye grass, grass mix, alternaria, dog hair, aspergillus

‡‡ Any of egg white, egg yolk, salmon, tuna, peanuts

**Table S3. Associations of lung function outcomes at age 8 years in relation to traffic density within a 50m radius of home adjusted for potential confounders and stratified by fish oil supplementation**

|  | **no fish oil supplementation** | | | | **Fish oil supplementation** | | | | **p-value for interaction** |
| --- | --- | --- | --- | --- | --- | --- | --- | --- | --- |
|  | **N** | **Mean Difference** | **95% CI** | **p-value** | **N** | **Mean Difference** | **95% CI** | **p-value** |  |
| log(FEV1 pre bronchodilator (L)) | 187 | -0.03 | -0.05-0.00 | 0.090 | 188 | 0.02 | -0.01-0.05 | 0.196 | 0.192 |
| log(FEV1 post bronchodilator (L)) | 184 | -0.01 | -0.04-0.02 | 0.459 | 188 | 0.02 | -0.01-0.05 | 0.150 | 0.156 |
| log (FVC pre bronchodilator (L)) | 184 | -0.02 | -0.05-0.01 | 0.194 | 185 | 0.02 | -0.01-0.05 | 0.118 | 0.364 |
| log (FVC post bronchodilator (L)) | 181 | -0.01 | -0.04-0.02 | 0.608 | 185 | 0.02 | -0.01-0.05 | 0.183 | 0.666 |
| FEV1/FVC ratio pre bronchodilator | 184 | -0.01 | -0.02-0.01 | 0.442 | 185 | 0 | -0.02-0.01 | 0.598 | 0.441 |
| FEV1/FVC ratio post bronchodilator | 181 | 0 | -0.02-0.01 | 0.557 | 185 | 0 | -0.01-0.01 | 0.918 | 0.388 |
| pre Peak Expiratory Flow (PEF) | 183 | -0.46 | -10.40-9.48 | 0.927 | 183 | -7.74 | -17.92-2.45 | 0.135 | 0.185 |
| post Peak Expiratory Flow (PEF) | 181 | 3.34 | -6.39-13.07 | 0.498 | 181 | -9.84 | -20.39-0.71 | 0.067 | 0.709 |
| Pre forced expiratory flow at 50% vital capacity (FEF_50_) | 183 | -0.05 | -0.19-0.08 | 0.446 | 183 | -0.01 | -0.16-0.14 | 0.898 | 0.562 |
| Post forced expiratory flow at 50% vital capacity(FEF_50_) | 181 | -0.03 | -0.16-0.10 | 0.670 | 181 | -0.13 | -0.28-0.02 | 0.097 | 0.817 |
| Pre forced expiratory flow at mid-expiratory phase(FEF_25-75_) | 183 | -0.03 | -0.14-0.09 | 0.665 | 183 | -0.05 | -0.17-0.06 | 0.357 | 0.277 |
| Post forced expiratory flow at mid-expiratory phase(FEF_25-75_) | 181 | 0 | -0.11-0.11 | 0.949 | 181 | -0.11 | -0.23-0.01 | 0.071 | 0.492 |

All models are adjusted for age at spirometry, height at spirometry, weight at spirometry, sex, father’s education, mother’s education, environmental tobacco smoke exposure, breastfed to 6 months, any dog owned by 5 or 8 years, any cat owned by 5 or 8 years, maternal smoking in pregnancy, gas cooking at home.

Interaction term of fish oil supplementation with traffic density was added as a separate term into a random intercept Poisson model.

Mean difference, Relative Risks (RRs) and 95 % confidence intervals (95% CI) represent increase in risk per unit increase in traffic density, representing 100m local road or 33.3m of motorway within 50m of home.

N=number of children.

**Table S4. Repeated measures analysis of combined age 5 and 8 years: Associations of selected allergic and respiratory outcomes in relation to traffic density within a 50m radius of home adjusted for potential confounders stratified by fish oil supplementation.**

|  | **no fish oil supplementation** | | | | **fish oil supplementation** | | | |  |
| --- | --- | --- | --- | --- | --- | --- | --- | --- | --- |
|  | **N** | **Mean difference** | **95% CI** | **p-value** | **N** | **Mean difference** | **95% CI** | **p-value** | **p-value for interaction** |
| Total IgE ( kU/L) | 143 | -0.023 | -0.28-0.24 | 0.860 | 148 | -0.11 | -0.36-0.15 | 0.414 | 0.786 |
| **Questionnaire variables** |  | **RR** |  |  |  | **RR** |  |  |  |
| Doctor diagnosed asthma | 184 | 1.05 | 0.80-1.38 | 0.728 | 187 | 1.14 | 0.89-1.46 | 0.288 | 0.587 |
| Ever had doctor diagnosed asthma | 184 | 1.07 | 0.84-1.35 | 0.595 | 187 | 1.03 | 0.84-1.27 | 0.770 | 0.797 |
| Wheeze in the last 12 months | 184 | 1.15 | 0.88-1.50 | 0.312 | 187 | 1.00 | 0.79-1.26 | 0.988 | 0.437 |
| Wheeze& asthma in the last 12 months | 184 | 1.11 | 0.79-1.56 | 0.555 | 187 | 0.96 | 0.71-1.29 | 0.770 | 0.697 |
| Cough more than 4 times in the last 12 months | 184 | 1.16 | 0.90-1.50 | 0.250 | 187 | 1.11 | 0.88-1.38 | 0.384 | 0.733 |
| Doctor diagnosed Eczema | 184 | 1.09 | 0.82-1.45 | 0.549 | 187 | 1.23 | 0.98-1.55 | 0.073 | 0.796 |
| Eczema in the last 12 months | 182 | 1.31 | 0.93-1.83 | 0.118 | 185 | 1.14 | 0.84-1.56 | 0.401 | 0.657 |
| Doctor diagnosed rhinitis | 184 | 1.08 | 0.69-1.72 | 0.729 | 187 | 1.98 | 1.40-2.80 | 0.0001 | 0.255 |
| **Positive Skin prick tests** |  |  |  |  |  |  |  |  |  |
| Any of 11 inhalant and food allergens | 175 | 1.20 | 1.00-1.45 | 0.052 | 181 | 0.92 | 0.74-1.14 | 0.445 | 0.072 |
| Inhalant allergen | 175 | **1.23** | **1.01-1.49** | **0.034** | 181 | 0.94 | 0.75-1.17 | 0.560 | 0.067 |
| Ingested allergen | 175 | 0.91 | 0.45-1.85 | 0.798 | 181 | 0.798 | 0.48-1.32 | 0.382 | 0.623 |
| House dust mite (HDM) | 175 | **1.37** | **1.09-1.72** | **0.007** | 181 | 1.04 | 0.83-1.31 | 0.714 | 0.066 |
| Alternaria tenuis | 175 | 0.93 | 0.56-1.52 | 0.758 | 181 | 0.75 | 0.46-1.20 | 0.225 | 0.192 |
| Grass mix | 175 | 1.18 | 0.75-1.84 | 0.471 | 181 | 0.82 | 0.50-1.32 | 0.408 | 0.384 |
| Rye Grass | 175 | 1.16 | 0.79-1.70 | 0.457 | 181 | 0.70 | 0.39-1.27 | 0.246 | 0.188 |
| **HDM sensitization** |  |  |  |  |  |  |  |  |  |
| IL-5 (>10 pg/ml) | 122 | **1.58** | **1.20-2.06** | **0.0009** | **134** | **0.72** | **0.54-0.97** | **0.0324** | **0.00015** |
| IL-10 (>10 pg/ml) | 120 | 1.15 | 0.90-1.48 | 0.270 | 129 | 0.91 | 0.79-1.04 | 0.156 | 0.0723 |

All models are adjusted for sex, father’s education, mother’s education, environmental tobacco smoke exposure, breastfed to 6 months, any dog owned by 5 or 8 years, any cat owned by 5 or 8 years, maternal smoking in pregnancy, gas cooking at home. Interaction term of fish oil supplementation with traffic density was added as a separate term into a random intercept Poisson model. Mean difference, Relative Risks (RRs) and 95 % confidence intervals (95% CI) represent increase in risk per unit increase in traffic density, representing 100m local road or 33.3m of motorway within 50m of home. N=number of children.

**Table S5. Repeated measures analysis of combined age 5 and 8 years: Associations of lung function measurements in relation to traffic density within a 50m radius of home adjusted for potential confounders and stratified by fish oil supplementation**.

|  | **no fish oil supplementation** | | | | **fish oil supplementation** | | | |  |
| --- | --- | --- | --- | --- | --- | --- | --- | --- | --- |
|  | **N** | **Mean Difference** | **95% CI** | **p-value** | **N** | **Mean Difference** | **95% CI** | **p-value** | **p-value for interaction** |
| log(FEV1 pre bronchodilator (L)) | 162 | -0.01 | -0.04-0.01 | 0.238 | 164 | -0.01 | -0.04-0.01 | 0.310 | 0.871 |
| log(FEV1 post bronchodilator (L)) | 160 | 0 | -0.02-0.02 | 0.997 | 163 | -0.01 | -0.03-0.02 | 0.638 | 0.711 |
| log (FVC pre bronchodilator (L)) | 161 | -0.01 | -0.04-0.01 | 0.397 | 162 | 0 | -0.03-0.02 | 0.879 | 0.904 |
| log (FVC post bronchodilator (L)) | 158 | 0.00 | -0.02-0.03 | 0.967 | 162 | 0 | -0.02-0.03 | 0.697 | 0.991 |
| FEV1/FVC ratio pre bronchodilator | 161 | -0.01 | -0.02-0.00 | 0.229 | 162 | -0.01 | -0.02-0.00 | 0.222 | 0.918 |
| FEV1/FVC ratio post bronchodilator | 158 | -0.01 | -0.02-0.00 | 0.307 | 162 | -0.01 | -0.02-0.00 | 0.099 | 0.94 |
| pre Peak Expiratory Flow (PEF) | 132 | -0.15 | -5.67-5.36 | 0.956 | 136 | -4.00 | -9.04-1.03 | 0.119 | 0.257 |
| post Peak Expiratory Flow (PEF) | 129 | 1.50 | -4.19-7.19 | 0.606 | 135 | -4.48 | -9.71-0.75 | 0.093 | 0.153 |
| Pre forced expiratory flow at 50% vital capacity (FEF_50_) | 132 | -0.03 | -0.13-0.06 | 0.503 | 136 | -0.04 | -0.13-0.05 | 0.347 | 0.826 |
| Post forced expiratory flow at 50% vital capacity(FEF_50_) | 129 | 0 | -0.10-0.10 | 0.985 | 135 | **-0.11** | **-0.20- -0.01** | **0.033** | 0.137 |
| Pre forced expiratory flow at mid-expiratory phase(FEF_25-75_) | 132 | -0.02 | -0.10-0.06 | 0.632 | 136 | -0.06 | -0.13-0.02 | 0.143 | 0.42 |
| Post forced expiratory flow at mid-expiratory phase(FEF_25-75_) | 129 | 0.01 | -0.08-0.10 | 0.769 | 135 | -0.1 | -0.18- -0.02 | 0.018 | 0.0585 |

All models are adjusted for age at spirometry, height at spirometry, weight at spirometry, sex, father’s education, mother’s education, environmental tobacco smoke exposure, breastfed to 6 months, any dog owned by 5 or 8 years, any cat owned by 5 or 8 years, maternal smoking in pregnancy, gas cooking at home.

Interaction term of fish oil supplementation with traffic density was added as a separate term into a random intercept Poisson model. Mean difference, Relative Risks (RRs) and 95 % confidence intervals (95% CI) represent increase in risk per unit increase in traffic density, representing 100m local road or 33.3m of motorway within 50m of home. N=number of children.

**Table S6. Repeated measures analysis of combined age 5 and 8 years in atopic children: Associations of selected respiratory outcomes in relation to traffic density within a 50m radius of home stratified by fish oil supplementation**

|  | **no fish oil supplementation** | | | | **fish oil supplementation** | | | | **p-value for interaction** |
| --- | --- | --- | --- | --- | --- | --- | --- | --- | --- |
|  | **N** | **RR** | **95%CI** | **p-value** | **N** | **RR** | **95%CI** | **p-value** |  |
| Doctor diagnosed asthma | 69 | 1.26 | 0.91-1.74 | 0.169 | 77 | 1.21 | 0.95-1.54 | 0.117 | 0.671 |
| Ever had doctor diagnosed asthma | 69 | 1.18 | 0.92-1.53 | 0.197 | 77 | 1.21 | 0.97-1.51 | 0.085 | 0.910 |
| Wheeze in the last 12 months | 69 | 1.28 | 0.91-1.79 | 0.157 | 77 | 0.99 | 0.77-1.27 | 0.942 | 0.338 |
| Wheeze& asthma in the last 12 months | 69 | 1.25 | 0.85-1.84 | 0.255 | 77 | 1.05 | 0.79-1.40 | 0.742 | 0.489 |
| Cough more than 4 times in the last 12 months | 69 | 1.22 | 0.88-1.69 | 0.223 | 77 | 1.31 | 1.03-1.67 | 0.028 | 0.585 |
| Doctor diagnosed Eczema | 69 | 1.19 | 0.85-1.68 | 0.312 | 77 | **1.47** | **1.13-1.90** | **0.003** | 0.350 |
| Eczema in the last 12 months | 68 | **1.57** | **1.01-2.45** | **0.046** | 77 | 1.26 | 0.93-1.70 | 0.130 | 0.498 |
| Doctor diagnosed rhinitis | 69 | 1.16 | 0.60-2.27 | 0.657 | 77 | 1.94 | 1.11-3.38 | 0.019 | 0.424 |

All models are adjusted for sex, father’s education, mother’s education, environmental tobacco smoke exposure, breastfed to 6 months, any dog owned by 5 or 8 years, any cat owned by 5 or 8 years, maternal smoking in pregnancy, gas cooking at home

Interaction term of fish oil supplementation with traffic density was added as a separate term into a random intercept Poisson model. Mean difference, Relative Risks (RRs) and 95 % confidence intervals (95% CI) represent increase in risk per unit increase in traffic density, representing 100m local road or 33.3m of motorway within 50m of home. N=number of children.

**Table S7 Repeated measures analysis of combined age 5 and 8 years in atopic children. Associations of lung function measurements in relation to traffic density within a 50m radius of home adjusted for potential confounders and stratified by fish oil supplementation**

|  | **no fish oil supplementation** | | | | **fish oil supplementation** | | | |  |  |  |
| --- | --- | --- | --- | --- | --- | --- | --- | --- | --- | --- | --- |
|  | **N** | **Mean Difference** | **95% CI** | **p-value** | **N** | **Mean Difference** | **95% CI** | **p-value** | **Mean Difference** | **95% CI** | **p-value for interaction** |
| log(FEV1 pre bronchodilator (L)) | 60 | 0 | -0.04-0.03 | 0.931 | 67 | -0.01 | -0.06-0.04 | 0.706 | -0.01 | -0.08-0.05 | 0.644 |
| log(FEV1 post bronchodilator (L)) | 60 | 0.03 | -0.02-0.08 | 0.286 | 67 | -0.01 | -0.05-0.04 | 0.732 | -0.04 | -0.10-0.03 | 0.262 |
| log (FVC pre bronchodilator (L)) | 59 | 0.02 | -0.03-0.06 | 0.500 | 67 | 0 | -0.06-0.05 | 0.914 | -0.02 | -0.10-0.05 | 0.502 |
| log (FVC post bronchodilator (L)) | 58 | 0.05 | -0.01-0.11 | 0.096 | 66 | -0.02 | -0.06-0.03 | 0.465 | -0.06 | -0.13-0.01 | 0.097 |
| FEV1/FVC ratio pre bronchodilator | 60 | 0 | -0.04-0.03 | 0.795 | 67 | -0.01 | -0.05-0.03 | 0.647 | -0.01 | -0.07-0.04 | 0.707 |
| FEV1/FVC ratio post bronchodilator | 60 | 0.01 | -0.03-0.06 | 0.525 | 67 | -0.01 | -0.05-0.03 | 0.645 | -0.03 | -0.08-0.03 | 0.327 |
| pre Peak Expiratory Flow (PEF) | 59 | 0.01 | -0.03-0.05 | 0.582 | 67 | 0 | -0.04-0.04 | 0.829 | -0.02 | -0.08-0.03 | 0.439 |
| post Peak Expiratory Flow (PEF) | 58 | 0.03 | -0.02-0.07 | 0.210 | 66 | -0.01 | -0.05-0.02 | 0.408 | -0.05 | -0.10-0.01 | 0.102 |
| Pre forced expiratory flow at 50% vital capacity (FEF_50_) | 59 | -0.02 | -0.04-0.00 | 0.060 | 67 | 0 | -0.02-0.02 | 0.956 | 0.02 | -0.01-0.04 | 0.239 |
| Post forced expiratory flow at 50% vital capacity(FEF_50_) | 58 | -0.02 | -0.04-0.00 | 0.070 | 66 | 0.01 | -0.01-0.02 | 0.517 | 0.02 | -0.00-0.05 | 0.087 |
| Pre forced expiratory flow at mid-expiratory phase(FEF_25-75_) | 49 | -2.9 | -12.3-6.51 | 0.546 | 54 | -9.8 | -20.12-0.53 | 0.063 | -5.08 | -19.07-8.91 | 0.476 |
| Post forced expiratory flow at mid-expiratory phase(FEF_25-75_) | 47 | -1.11 | -11.9-9.76 | 0.842 | 53 | -11.05 | -22.14-0.04 | 0.051 | -8.9 | -23.89-6.08 | 0.244 |

All models are adjusted for age at spirometry, height at spirometry, weight at spirometry, sex, father’s education, mother’s education, environmental tobacco smoke exposure, breastfed to 6 months, any dog owned by 5 or 8 years, any cat owned by 5 or 8 years, maternal smoking in pregnancy, gas cooking at home.

Interaction term of fish oil supplementation with traffic density was added as a separate term into a random intercept Poisson model. Mean difference, Relative Risks (RRs) and 95 % confidence intervals (95% CI) represent increase in risk per unit increase in traffic density, representing 100m local road or 33.3m of motorway within 50m of home. N=number of children.
